# Larviciding for malaria control and elimination in Africa

**DOI:** 10.1186/s12936-024-05236-y

**Published:** 2025-01-15

**Authors:** Gretchen Newby, Prosper Chaki, Mark Latham, Dulcisária Marrenjo, Eric Ochomo, Derric Nimmo, Edward Thomsen, Allison Tatarsky, Elijah O. Juma, Michael Macdonald

**Affiliations:** 1 Malaria Elimination Initiative, Institute for Global Health Sciences, University of California, 550 16 St, San Francisco, CA 94158 USA; 2 Pan-African Mosquito Control Association, Kenya Medical Research Institute, Mbagathi Road, PO Box 4445-00100, Nairobi, Kenya; 3 Ifakara Health Institute, 67M3+GM3, Kiko Ave, Dar Es Salaam, Tanzania; 4 Manatee County Mosquito Control District, 1420 28Th Ave E, Ellenton, FL 34222 USA; 5 Programa Nacional de Controlo da Malária, Ministério da Saúde, Maputo, Mozambique; 6 Center for Global Health Research, Kenya Medical Research Institute, PO Box 1578-40100, Kisumu, Kenya; 7 RBM Partnership Vector Control Working Group, Chem du Pommier 40, 1218 Le Grand-Saconnex, Switzerland; 8 Liverpool School of Tropical Medicine, IVCC, Pembroke Place, Liverpool, L3 5QA UK

**Keywords:** Malaria, Larval source management, Larviciding, Africa, Regional collaboration, Global policy

## Abstract

**Background:**

Global progress toward malaria elimination and eradication goals has stagnated in recent years, with many African countries reporting increases in malaria morbidity and mortality. Insecticide-treated nets and indoor residual spraying are effective, but the emergence and increased intensity of insecticide resistance and the challenge of outdoor transmission are undermining their impact. New tools are needed to get back on track towards global targets. This Perspective explores the major challenges hindering wider-scale implementation of larviciding in Africa and identifies potential solutions and opportunities to overcome these barriers.

**Larviciding in Africa: overview, challenges, and solutions:**

Larviciding is a valuable vector control tool with strong potential for regional scale-up. There is considerable evidence of its effectiveness, and the World Health Organization (WHO) recommends it as a supplemental intervention. However, malaria programmes hoping to implement larviciding face significant barriers, including (1) poor global technical, policy, and funding support; (2) fragmented implementation and experience; (3) high complexity of delivery and impact evaluation; and (4) limited access to the full range of WHO prequalified larvicide products. Strategic barriers related to global policy and donor hesitancy can be overcome through a coordinated demonstration of cost-effectiveness. Technological advancements and strengthened operational capacity have already overcome technical barriers related to larvicide delivery, targeting, coverage, and evaluation. Developing a Community of Practice platform for larviciding has strong potential to consolidate efforts, addressing the challenge of fragmented implementation and experience. Such a platform can serve as a resource center for African malaria programmes, collating and disseminating technical guidance, facilitating the exchange of best practices, and aiding malaria programmes and partners in designing and evaluating larviciding projects.

**Conclusion:**

The global shift toward targeted and adaptive interventions enables the incorporation of larviciding into an expanded vector control toolbox. As more African countries implement larvicide programmes, establishing a regional Community of Practice platform for exchanging experiences and best practices is necessary to strengthen the evidence base for cost-effective implementation, advocate for support, and inform policy recommendations, thus supporting Africa’s progress toward malaria elimination.

## Background

After nearly two decades of impressive reductions in malaria morbidity and mortality, global progress slowed in 2016 and has remained stagnant. This trend has been uneven, with several low burden countries continuing their trajectory towards malaria elimination as high burden countries, mainly in sub-Saharan Africa, have seen an increase in cases and deaths [1]. There are multiple contributing factors: funding for malaria has plateaued; parasite and vector adaptations have diminished the impact of antimalarial drug regimens, indoor residual spraying (IRS), and insecticide-treated nets (ITNs); mass population displacement arising from conflict and extreme weather events has disrupted access to healthcare among vulnerable populations; disruptions in the malaria commodity supply chain due to the Covid-19 pandemic; and health systems and governments have been challenged by multiple competing priorities for limited resources [2]. Despite efforts to catalyze renewed progress and rethink the global approach to malaria control [3, 4], this stagnation has persisted.

Recent research has shown that global eradication of malaria can be achieved, but getting back on track requires innovation in both technology and practice [5]. Existing tools must be strategically deployed with greater efficiency using new, data-driven approaches, and the research and development pipeline for new tools needs continued investment [5, 6]. The malaria community has made considerable progress on both. Global policy has shifted from universal coverage of just one or two interventions to a broad, diverse set of targeted and tailored approaches based on district- or subdistrict-level stratification of malaria transmission, broadly referred to as sub-national tailoring [7]. In addition to advances in vaccines and gene drive technologies [8, 9], new insecticide active ingredients, formulations, and combinations for ITNs are already showing impact [10, 11], as are new vector control tools and approaches including spatial emanators, eave tubes, and housing modifications [12–14].

The potential of larval source management (LSM) to complement the impact of ITNs and IRS and strengthen the vector control toolbox is increasingly being recognized. LSM has been underused as a modern-era malaria control tool in sub-Saharan Africa, despite its historical use in a range of eco-epidemiological settings, its critical role in achieving and maintaining malaria elimination, and extensive evidence of its importance for control of nuisance mosquitoes and mosquito-borne diseases other than malaria [15–20]. In 2013, the World Health Organization (WHO) acknowledged LSM’s role as a supplementary measure to IRS and ITNs, and in its most recent guidelines, WHO conditionally recommended the use of larviciding, albeit in restricted settings such as urban areas [7, 21]. In a more explicit endorsement of its utility, the 2022 *Global framework for the response to malaria in urban areas* featured LSM as the recommended approach for malaria prevention, including in the context of invasive *Anopheles stephensi* control [22]. This growing global support for LSM is promising. However, major donors have not invested in LSM and national malaria programmes eager to include it as a routine intervention in their strategic plans have had to rely on limited domestic funding, which, in many cases, has restricted the scope and duration of LSM activities [23–25].

The role of LSM must be reconsidered and reprioritized in light of current challenges to reducing malaria transmission. This perspective summarizes the current landscape of LSM in Africa with a focus on larviciding, examines the barriers to implementing larviciding as a key component of the malaria vector control toolbox, and suggests solutions to overcoming those barriers.

## Overview of larval source management

LSM reduces adult mosquito density by targeting larval and pupal stages in their aquatic habitats. This can be done through environmental modification or manipulation of larval habitats, introduction of natural predators like *Gambusia affinis*, or application of microbial or chemical larvicides and insect growth regulators to water bodies [21]. LSM in its various forms has been used for malaria control for over 100 years—some of the first examples date back to the early 1900s in the Indo Pacific [26, 27]—and has been amply documented in published literature [16, 28]. It was the primary approach to malaria control in the decades leading up to the Global Malaria Eradication Programme (GMEP), but set aside in the 1950s in favour of IRS which was deemed cheaper, simpler to execute in mass campaigns through well-established channels and infrastructure, and did not require extensive planning, training, or in-depth knowledge of the environment or vector ecology [29]. As detailed below, the current toolbox of larval control products, application equipment and information management are far more effective, scalable, and less environmentally harmful than the original methods of spent-diesel fuel for oiling and the broadly toxic Paris Green (Copper acetoarsenite).

Meanwhile, the United States relied heavily on environmental management and water engineering projects to reduce malaria transmission in the 1930–40s. Some strategies had negative environmental consequences through destruction of wetlands ecosystems. But like the evolution from Paris Green, current strategies call for close collaboration between ecologists, mosquito control, regulators and communities for the common goals of preserving wetlands while addressing the public health concerns [30]. After achieving elimination in 1951, local mosquito management programmes in the US continued to experiment with LSM before adopting what would become known as an integrated vector management (IVM) approach [29, 31, 32]. IVM is a rational decision-making process geared towards optimizing resources and improving efficacy, cost-effectiveness, ecological soundness, and sustainability of vector control interventions [33].

Modern mosquito control programmes in the Americas and Europe continue to implement IVM, with larviciding used alongside adulticiding where effective and appropriate [34–36]. Although these mosquito control programmes operate in a different ecological context than Africa, with different vectors, vector-borne diseases, and vector control tools, the principles of IVM are universal [37]. Notably, the success of these programmes is driven by some of the same factors that deterred the GMEP: extensive planning and management of human and financial resources, detailed mapping of the natural and constructed environment, and rigorous entomological surveillance to understand vector ecology, monitor vector density and behaviour, and inform decision-making [38].

## Challenges to implementing larviciding in Africa

Targeting adult stage mosquitoes through a combination of IRS and ITNs has been the cornerstone of malaria vector control in Africa since the 1990s. The operational focus has recently broadened, particularly in the past decade as vector biting and resting has shifted from predominantly indoor to increasingly outdoor, due to a combination of changes in vector behaviour and proportional shifts in species comprising vector populations. African malaria programmes have been quick to identify the need for adaptive approaches to regain momentum and are increasingly incorporating larviciding into their local targeting and tailoring strategies [39]. Yet, programmes aspiring to scale up implementation of larviciding face significant barriers, including (1) poor global technical, policy, and funding support (2) fragmented implementation and experience, (3) high complexity of delivery and impact evaluation, and (4) limited access to the full range of WHO prequalified larvicide products.

## Poor global technical, policy, and funding support

After the re-start of global malaria control and elimination efforts in the 1990s [40, 41], the focus for scaling up vector control was entirely on IRS and ITNs, with no mention of LSM [42]. Twenty-five years later, WHO’s updated guidelines include a conditional recommendation for larviciding but note a lack of quality evidence of its impact [7]. The recommendation is a step in the right direction, but the tepid and cautionary language on low-certainty evidence and its role only as a supplementary measure has stifled broad support by major policymakers and donors [43, 44].

Like the tools, the policy recommendations are evolving. With technical advances in targeting, delivery and evaluation, and improved Standard Operating Procedures and sharing of best practices, larviciding can demonstrate to funders and policy-makers its value as a core intervention. It is important to note however, that larviciding is not universally applicable in every malaria transmission context, but can depend upon the ecology and extent of the larval habitats in relation to the proximity and density of the human population to be protected. As discussed below, some may argue that the “Gold Standard” cluster randomized controlled trial (cRCT) design does not provide external validity in the case of a complex, environmentally-driven process like LSM. As in the Water, Sanitation and Hygiene (WASH) sector, other mixed-methods evaluations may be more appropriate [45].

Recent larviciding implementation in Africa has mainly been funded domestically or by the private sector [39]. However, larviciding is currently too expensive to deliver as a routine intervention without considerable financial support, thus limiting the scope, duration, impact, and sustainability of each project. The US President’s Malaria Initiative (PMI) has started supporting short-term pilots in a few countries, which is a positive development [46, 47]. Support from other major donors like the Global Fund to Fight AIDS, Tuberculosis and Malaria would alleviate the financial burden on malaria programmes and allow programme managers to focus instead on capacity-strengthening, high-quality implementation, and local evidence generation. Robust cost-effectiveness data comparing larviciding to other interventions is needed to influence policy decisions and attract funding.

## Fragmented implementation and experience

Managing an effective larviciding programme requires more technical expertise and ecological understanding than ITN implementation and can be as logistically challenging as IRS. Before the mid-2000s, there were very few IRS programmes in Africa with large-scale implementation experience. Primarily through the capacity-strengthening efforts of PMI, IRS was massively scaled up to 14 countries, with 28,000 trained spray personnel and more than 5 million houses sprayed in 2022 [48]. In the process, the malaria community has made tremendous improvements to the IRS management cycle, strengthening collective knowledge and practice in planning, training, targeting, application, and monitoring and evaluation of IRS operations. While IRS has been reduced in several countries for a variety of financial and operational reasons, it can serve as a model for rapidly expanding capacities and best practices.

There have been no such concerted efforts for larviciding. While there have been some large and successful programmes in Dar es Salaam and Khartoum [49, 50], much of the recent larviciding has been implemented either as a programmatic intervention or in small-scale pilot projects with few mechanisms for learning and sharing best practices. The lack of standardized approaches to larviciding implementation and evaluation makes it difficult to compare results across different contexts, hindering the accumulation of evidence needed to inform best practices. The RBM Partnership to End Malaria Vector Control Working Group (RBM VCWG) [51] conducted a landscaping survey of LSM projects active between the 2021–2023 calendar years. Of the 48 African countries surveyed, 23 responded; all 23 reported carrying out LSM and, of those, 15 conducted larviciding—a likely undercount, considering the 25 non-responses (unpublished data, 2022). The survey revealed that programmes use a narrow range of products and various methods and protocols for targeting, delivery, monitoring, and evaluation. This limited, piecemeal approach hinders the establishment of best practices and the consolidation of evidence needed to demonstrate impact and inform future policy changes.

## High complexity of delivery and impact evaluation

Unlike IRS, there is no generalizable approach to larviciding. Each vector species, ecological setting, and programmatic context requires a locally tailored solution. Larviciding will not be appropriate everywhere, and success requires a more targeted approach than ITN or IRS delivery. In Africa, larviciding has played an important role in controlling malaria in the arid Sahel region where many larval habitats are seasonal and manmade [50, 52], in southern Africa, where the cool, dry season reduces larval habitats and extends the development period of immature mosquito stages [53], and in urban areas where the ratio of human population density to larval habitats is high and habitats are easier to identify and access [50, 54, 55]. Knowing when and where it will likely work, and where it will likely not, requires thorough, up-to-date epidemiological and entomological surveillance data, knowledge of the local environment and vector ecology, and awareness of historical and contemporary successes and failures.

Unfortunately, the technical expertise necessary to successfully navigate these complexities and implement an effective larviciding programme began to be deprioritized during the GMEP era,when rigorous execution of IRS spraying campaigns shifted the role of malariologists from field scientists and problem-solvers to project managers [56]. A one-size-fits-all approach has persisted in the malaria vector control community for decades, emphasizing universal coverage of IRS and ITNs, devaluing larviciding and other LSM approaches, and hindering the development of collective expertise and capacity. More recently, the growth of molecular surveillance and genomics may have further reduced expertise in field-based vector ecology. However, this trend is showing promising signs of change. As the 2021 WHO/Harvard exercise on “Rethinking Malaria” recommends, there needs to be increased investment in the health workforce at all levels; visibility and use of reliable and real-time data, knowledge and information; and greater attention should be given to innovation and problem-solving [57].

Even now, with the new emphasis on local targeting and tailoring in place of universal coverage, the complexity of larviciding can be a deterrent to major donors because of mixed messages on its effectiveness as well as its fragmented implementation. In addition, malaria donors prioritize economic efficiency through commodity procurement and delivery. After decades of implementation, the efficiency of IRS and ITNs is easily measured by standardized indicators based on unit cost, programme implementation costs, population at risk, and achieved coverage [58]. The primary indicator used for larviciding has been cost per person protected per year, and a 2011 costing analysis of larviciding in different African contexts determined that costs compared favorably with those for IRS and ITNs, especially in areas with moderate and focal malaria transmission where mosquito larval habitats are accessible and well-defined [59]. However, an updated and broader set of standardized indicators that facilitate better comparison with current IRS and ITN implementation is needed. Similarly, there are no standardized indicators or methods such as bioassays and entomological sampling for monitoring and evaluation of *Anopheles* control to ensure that high-quality delivery of larvicides is maintained and has an impact.

The WHO prioritizes evidence generated through randomized controlled trials (RCTs) over contextual evidence when evaluating intervention impact [60]. However, because larviciding programmes are so context-specific, RCTs with epidemiological endpoints may have internal validity for that time and place, but results cannot be applied to other ecological or programmatic contexts. Other environmentally-driven processes and interventions, such as those in the water, sanitation and hygiene sector, are moving beyond the RCT “gold standard” based exclusively on epidemiological endpoints in favor of a mixed methods research toolkit for evaluation [61]. A mixed methods approach that considers a broader range of available evidence, including entomological endpoints, implementation indicators, and possibly modelling is more appropriate for evaluating impact of larviciding programmes, as recently demonstrated in Sudan [62].

## Limited access to the full range of WHO prequalified larvicide products

Malaria programmes should select the larvicides or larvicide combinations most appropriate for target habitat and mosquito species based on proven efficacy, ease of use, residual activity, cost, and public acceptance. The WHO’s prequalification framework addresses some of these considerations by ensuring quality, safety, and appropriateness for use in lower- and middle-income countries [63]. However, there are still significant barriers to product accessibility.

The WHO has prequalified 21 larviciding products [64], but the RBM VCWG landscaping exercise revealed that a limited number of those products are in use in Africa (Table 1). The most commonly used larvicide in Africa is *Bacillus thuringiensis israelensis* (Bti), although only six countries use WHO-prequalified products, which are dry formulations that have greater stability in local ambient temperatures [65]. Six other countries are using a liquid Bti formulation manufactured in Tanzania that is bulkier and, despite having a lower price point than prequalified products, is more expensive to transport and store and may have reduced efficacy at local ambient temperatures.

**Table 1:** Larvicides in use in Africa*

Larvicide	Type	# products	# manufacturers	# African countries using
WHO prequalified larvicides				
Temephos	Organophosphate	3	2	1
Pirimiphos methyl	Organophosphate	1	1	-
Polydimethylsiloxane	Monomolecular film	1	1	1
Diflubenzuron	Insect growth regulator	3	1	-
Novaluron	Insect growth regulator	1	1	-
Pyriproxyfen	Insect growth regulator	3	2	-
* Bacillus thuringiensis israelensis* (Bti)	Bacterium (dry formulation)	2	1	6
* Bacillus sphaericus*	Bacterium	1	1	-
Spinosad	Bacterium	6	2	-
Other larvicides (not prequalified)				
Methoprene	Insect growth regulator	3	1	1
Bti	Bacterium (liquid formulation)	1	1	6

^*^Source: RBM Vector Control Working Group landscaping survey, 2022

While non-prequalified products may gain national registration, the major international donors, Global Fund and PMI, only procure pre-qualified products. This compounds the problem of access to financing to support scale-up of larviciding operations in endemic countries.

Limited prequalified product usage in Africa can be explained in part by the fact that these larviciding products were originally created for the US market—where local mosquito control programmes generally have the human and financial resources to apply larvicide weekly—and they have limited residual activity. The advantages of residual products (> 30 days versus 7–14 days for non-residual formulations) are significant as they allow trained field personnel to cover larger areas more efficiently and effectively with fewer applications. For minimally-resourced malaria programmes, there are clear benefits conferred by larviciding products with extended residual control: fewer field visits and smaller cohorts of spray operators requiring training and equipment. However, despite the efficiencies gained in programme operations over the course of the malaria transmission season, higher upfront product costs can be a deterrent, leading programme managers to either select cheaper options, even when their residual activity is known to be insufficient, or bypass larviciding altogether. In general, Bti formulations are ideal from the standpoint of mosquito specificity, environmental compatibility, and human safety, but they do not provide extended residual control and have reduced efficacy in larval habitats high in organics, such as polluted waters, increasing the possibility of larvicide failure. Thus, using Bti alone in these conditions will not have as much impact as deploying a combination of products with different residual activities and modes of action, which may enhance effectiveness and extend the lifespan of the respective products.

Finally, despite a wide variety of larvicide products already in the market and in development, incentivizing manufacturers and distributors to pay to register and stock larvicide products in Africa’s currently limited and fragmented marketplace is a major challenge. These costs are prohibitive when there is no guarantee that larvicide applications will be implemented or continued yearly as part of national malaria strategies or that the larvicide product market will continue to expand. Manufacturing quality-assured larvicide locally can help reduce costs, and countries tend to support this approach because of the potential for local economic growth. However, mechanisms to link the major product manufacturers with local governments are not yet in place.

## Solutions

Some of the barriers to larvicide scale-up faced by malaria programmes, particularly those related to global policymaker and donor priorities, will require longer-term collaboration and advocacy efforts, as well as the elevation and prioritization of malaria programme managers’ perspectives and programmatic needs. In the meantime, progress in addressing challenges related to larvicide delivery, targeting, and coverage is already underway, and there are practical, near-term solutions that can be taken up by African malaria programmes and implementing partners to demonstrate where, when, and how larviciding can be an effective intervention. Progress in these areas will ultimately influence global policy and inform and strengthen advocacy for global funding support.

Larvicide application technology has vastly improved in recent years. Extensive field research conducted under different environmental conditions on larvicide formulation, droplet size, spray swath width and angle, and delivery method has informed the development of highly effective new tools and equipment. Wide-area application using this new technology, combined with improved targeting, particularly of Bti and other bacterial larvicides, allows for greater coverage of larval habitats, including cryptic or temporary habitats, overcoming the “few, fixed, findable” mandate of current WHO guidelines on larviciding [39]. Targeted aerial and ground-based applications of Bti with either backpack or vehicle-mounted sprayers and foggers have all proven effective against *Aedes* and *Culex* mosquito species in various settings and types of larval habitats. They could potentially be effective against *An. stephensi,* which shares some ecological similarities with *Aedes* vectors [66–69]. In a densely-populated urban setting in Puerto Rico where access to outdoor private property is limited, vehicle-mounted spraying successfully deposited Bti in a range of open and covered locations, including under vegetation and man-made structures, and effectively suppressed dengue transmission [69]. Drone-based delivery of bacterial larvicides for malaria control in rice fields has recently been piloted successfully in Madagascar and Rwanda [47, 70], and it is used routinely in Europe and the United States.

There is ample literature on the use of remote sensing for improved targeting of larval habitats for vector-borne disease control [71–73], and more recently, there have been tremendous advances in the accessibility, use, and cost-efficiency of remote sensing, geographical information systems, artificial intelligence, and mobile technology to identify, map, and target larval habitats. A review on the role of satellite data for malaria revealed a wealth of publicly available, high-resolution information on human and environmental factors (e.g., population movement, agriculture, deforestation, water management) that influence malaria transmission, and user-friendly platforms have improved the accessibility of this data [74]. Emerging technologies like drones equipped with high-resolution cameras and AI-powered image analysis can significantly improve the identification and mapping of mosquito larval habitats. These tools can enhance the precision and efficiency of larviciding operations, particularly in hard-to-reach areas. Many African malaria programmes are using these technologies to guide decision-making and targeting, including for larviciding (Table 2).

**Table 2:** Examples of new technologies for larval habitat identification and targeting in use in Africa

Name	Description	Country
Zzapp Malaria [75, 76]	Artificial intelligence identifies transmission hotspots using satellite imagery-based topographical maps, optimizes malaria elimination interventions and strategies, conveys simple instructions to field users via mobile app	Ethiopia Ghana Mozambique São Tomé and Príncipe Zanzibar
Google Earth Engine vector suitability mapping [77]	Cloud-based, continuous fine-resolution geospatial analytical tools used to develop an environmental suitability model considering water resources, flow accumulation areas, precipitation, temperature, vegetation, and land cover; model can be parameterized for different mosquito species, temporal periods and geographical boundaries	Malawi
Mosquito Alert, iNaturalist, GLOBE Observer's Mosquito Habitat Mapper and Land Cover [78]	Integration of citizen science platforms in which members of the general public upload images of mosquitoes and larval habitats to mobile apps to standardize and combine datasets, develop artificial intelligence identification software, and establish a global surveillance system for mosquito-borne disease	Global
Spatial Intelligence System (using drone-based imagery) [79, 80]	Low-cost unmanned aerial vehicles record high-resolution, georeferenced images of water bodies, access routes, adjacent households, and land cover; resulting orthomosaics are manually interpreted to delineate water body location and size; uploaded images merged with descriptions of habitats and larvicide treatment history in Zzapp Malaria platform; Zzapp smartphone app used to locate habitats on the ground and assess accuracy of mapping and treatment data	Zanzibar
Remote sensing and risk mapping [52, 81]	High spatial and spectral resolution data from satellite imagery is used to identify prediction parameters (e.g., land temperature, rainfall, vegetation cover, surface water); parameters fed into prediction models are used to generate risk maps that guide the targeting of most productive larval habitats	Burkina Faso

Regarding product availability, generating comparative product and implementation cost data and establishing a set of indicators appropriate for the African context that facilitates a fair evaluation of product cost-effectiveness is essential to help guide product choice, determine demand, and shape the larvicide market. Industry partner engagement, incentivization schemes, and exploration of public–private partnerships, as have been shown successful for IRS and ITNs, can also help expand market opportunities in Africa. In the near term, multiple methoprene formulations with extended residual activity are available but not yet in the WHO prequalification pipeline. Other products in development have a residual impact of up to 6 months, which will be particularly important for controlling invasive *An. stephensi* in urban container habitats [82].

Development of a “Community of Practice” platform for larviciding, and LSM more broadly, has strong potential to address the challenge of fragmented implementation and experience and the lack of standardized indicators and an evaluation framework to measure local impact. Similar platforms already exist; for example, the On-Line Resource Exchange Network for Entomology, managed by the Asia Pacific Malaria Elimination Network, is a platform for vector biologists in the Asia Pacific region to access and exchange information and resources for managing malaria and other mosquito-borne public health threats [83]. A Community of Practice platform can serve as a resource center for African malaria programmes, supporting collation and dissemination of global and regional technical guidance (e.g., on larviciding products, indicators, study design, comparative cost analyses, impact evaluations), providing summaries of current and planned research-based and programmatic larviciding implementation, facilitating the exchange of best practices, and aiding programme managers and partners in designing and evaluating larviciding projects using a standardized impact evaluation framework.

The Pan-African Mosquito Control Association (PAMCA) is ideally placed to establish and manage a dedicated Community of Practice platform for LSM in the African region. Since its launch in 2012, PAMCA has rapidly expanded and has chapters in 18 countries, providing a forum for entomology and vector control across various disciplines [84]. Because of its widespread presence in the region, PAMCA is ideally suited to convene and oversee country-led dialogues that promote location-specific evidence generation and provide technical support for operational planning and implementation initiatives at the subnational level. PAMCA can use its convening power to advocate for community-owned, sustainable approaches to implementing larviciding and other LSM interventions, empowering affected communities to be proactive in decision-making. In addition, PAMCA has close ties to the American Mosquito Control Association and its extensive LSM resources [85, 86] and it can leverage the expertise, exchange of best practices, and convening capacity of the RBM VCWG and its LSM Task Force, currently co-led by a PAMCA senior officer, to coordinate with countries who have not yet established a PAMCA chapter [87].

PAMCA and its partners can use the data and resources collated on the Community of Practice platform to engage with the WHO, Global Fund, PMI, and other global partners and amplify the voices of national malaria programmes, advocating for support, funding, and policy change. PAMCA and partners can also coordinate with product manufacturers on market shaping and facilitate linkages with governments interested in setting up local manufacturing for quality-assured, WHO-prequalified products. Finally, PAMCA and partners can promote investment in and support critical training and capacity-strengthening needs to build up an expanded cadre of public health entomologists, researchers, and skilled larvicide implementers in Africa.

## Conclusion

Conditions have never been more promising for including larviciding and other LSM approaches as key components of malaria control and elimination strategies in Africa. Wide-scale population movement, rapid urbanization and the spread of the invasive urban malaria vector *An. stephensi*, growing insecticide resistance, and changing vector behaviours have created a considerable gap in the region's coverage of IRS and ITNs [88]. The global shift away from universal coverage strategies to localized targeting and subnational tailoring sets the stage for the incorporation of larviciding into an expanded vector control toolbox, but the lack of global support and the current fragmented landscape creates a significant risk of poor-quality implementation and unintended negative outcomes which could undermine efforts to build widespread support for larviciding. With a dedicated Community of Practice platform in place, hosted by PAMCA and its strong network of partners, the African malaria community can begin to systematically and collaboratively address the needs and challenges outlined in this Perspective, ensuring that larviciding can fulfil its potential as a key vector control strategy and help the region get back on track toward malaria elimination.

## Data Availability

No datasets were generated or analysed during the current study.

## Abbreviations

Bti: *Bacillus thuringiensis israelensis*
GMEP: Global Malaria Eradication Programme
IRS: Indoor residual spraying
ITN: Insecticide-treated net
IVM: Integrated vector management
LSM: Larval source management
PAMCA: Pan-African Mosquito Control Association
PMI: US President’s Malaria Initiative
RBM VCWG: RBM Partnership to End Malaria Vector Control Working Group
WHO: World Health Organization
